# Bruton tyrosine kinase inhibitors in B-cell lymphoma: beyond the antitumour effect

**DOI:** 10.1186/s40164-022-00315-9

**Published:** 2022-09-22

**Authors:** Haoran Wang, Hao Guo, Jingyi Yang, Yanyan Liu, Xingchen Liu, Qing Zhang, Keshu Zhou

**Affiliations:** Department of Hematology, Affiliated Cancer Hospital of Zhengzhou University, Henan Cancer Hospital, No. 127 Dongming Road, Jinshui District, Zhengzhou, 450003 China

**Keywords:** BTK inhibitors, B-cell lymphoma, Immunomodulation, Tumour microenvironment, Combination therapy, Infection, Coronavirus disease 2019, Vaccination

## Abstract

Targeting B-cell receptor signalling using Bruton tyrosine kinase (BTK) inhibitors (BTKis) has become a highly successful treatment modality for B-cell malignancies, especially for chronic lymphocytic leukaemia. However, long-term administration of BTKis can be complicated by adverse on- and/or off-target effects in particular cell types. BTK is widely expressed in cells of haematopoietic origin, which are pivotal components of the tumour microenvironment. BTKis, thus, show broad immunomodulatory effects on various non-B immune cell subsets by inhibiting specific immune receptors, including T-cell receptor and Toll-like receptors. Furthermore, due to the off-target inhibition of other kinases, such as IL-2-inducible T-cell kinase, epidermal growth factor receptor, and the TEC and SRC family kinases, BTKis have additional distinct effects on T cells, natural killer cells, platelets, cardiomyocytes, and other cell types. Such mechanisms of action might contribute to the exceptionally high clinical efficacy as well as the unique profiles of adverse effects, including infections, bleeding, and atrial fibrillation, observed during BTKi administration. However, the immune defects and related infections caused by BTKis have not received sufficient attention in clinical studies till date. The broad involvement of BTK in immunological pathways provides a rationale to combine BTKis with specific immunotherapies, such as immune checkpoint inhibitor or chimeric antigen receptor-T-cell therapy, for the treatment of relapsed or refractory diseases. This review discusses and summarises the above-mentioned issues as a reference for clinicians and researchers.

## Background

B-cell lymphomas (BCLs), which include chronic lymphocytic leukaemia (CLL), diffuse large B-cell lymphoma, mantle cell lymphoma (MCL), Waldenstrom macroglobulinaemia (WM) and so on, are the most frequent haematologic malignancies. With the development of small-molecule targeted drugs such as Bruton’s tyrosine kinase (BTK) inhibitors (BTKis), B-cell lymphoma 2 inhibitors, and phosphoinositide 3-kinase (PI3K) inhibitors, treatment of BCL has undergone a tremendous change, especially for CLL. The use of BTKis, in particular, has benefited many patients, including those at high risk. The first-generation BTKi ibrutinib inhibits the proliferation and survival of B cells by irreversibly binding BTK C481 and blocking the B-cell receptor (BCR) signalling pathway. Ibrutinib also binds to other kinases, such as IL-2-inducible T-cell kinase (ITK), epidermal growth factor receptor (EGFR) [1], and TEC and SRC family kinases [2], to induce off-target effects. Although the antitumour activities of BTKis depend on both on-target and off-target effects, adverse events such as rashes, atrial fibrillation, and bleeding should not be ignored. The next-generation BTKis acalabrutinib, zanubrutinib, and orelabrutinib show higher selectivity and fewer off-target effects than ibrutinib, thereby limiting the adverse events profoundly. Till date, the inhibitors have been successfully approved for the treatment of relapsed/refractory (R/R) MCL and CLL. Recently, zanubrutinib has been approved for WM. Non-covalent BTKis, such as pirtobrutinib, vecabrutinib, and fenebrutinib, may have fewer adverse effects than the covalent BTK inhibitors and have shown promising safety profiles and efficacy in clinical trials.

Treatment with a BTKi potentially impacts both innate and adaptive immunity, including the number and function of various immune cells. BTK looks like a type of ‘Swiss Army knife’ and is expressed in myeloid and other innate immune cells. Thus, inhibition of BTK with a BTKi leads to changes in immune cell numbers. Additionally, different BTKis play pleiotropic roles (different effects on different types of target cells) in the regulation of immune cell function. Ibrutinib inhibits rituximab-dependent NK cell-mediated cytotoxicity, while acalabrutinib, orelabrutinib, and fenebrutinib have no effect on ITK- and NK cell-mediated antibody-dependent cellular cytotoxicity (ADCC), making them promising candidates for combination therapy with anti-CD20 antibodies. In addition, the influence of ibrutinib on T cells provides a rationale for the combined use of programmed cell death-ligand 1 (PD-L1) inhibitors, chimeric antigen receptor-T-cell (CAR-T) therapy, or bispecific antibody (BiAb) with a BTKi. Despite having no inhibitory effect on ITK, the next-generation BTKi acalabrutinib benefits CAR-T therapy; however, the exact mechanism remains unclear [3].

Tumour microenvironment (TME) plays a crucial role in the survival and growth of tumour cells by providing inhibitory or stimulatory signals, including BCR signals [4]. BTK can transmit and enhance molecular signals on the surface of various cells that communicate with the TME, via the Toll-like receptor (TLR) and FcγR on macrophages, dendritic cells, mast cells, and basophils [5]. In addition, BTK is a regulator of the NACHT, LRR, and PYD domain-containing protein 3 (NLRP3) inflammasome, which has been observed to be associated with various infections, including coronavirus disease 2019 (COVID-19), myocardial infarction, and other diseases such as Alzheimer’s disease and atherosclerosis [6]. Among the side effects, infections are associated with severity and poor prognosis in patients and are particularly complicated to manage. Moreover, BTKis have recently been shown to impact vaccination [7].

At present, the mechanisms of combination strategies of BTKis with specific immunotherapies are unclear. In addition, infections caused by the use of BTKis are common in clinical practice but have not attracted sufficient attention yet. Therefore, comprehensive exploration and understanding of these issues are urgently required. This review aimed to examine the pleiotropic effects of BTKis on the immune system and the potential combination strategies comprising BTKi and different immunotherapies, which may provide practical advice on the management of BTKi-related toxicity and shed light on optimal treatment options.

## BCR/BTK signaling in normal and malignant B cells

**Fig. 1:**
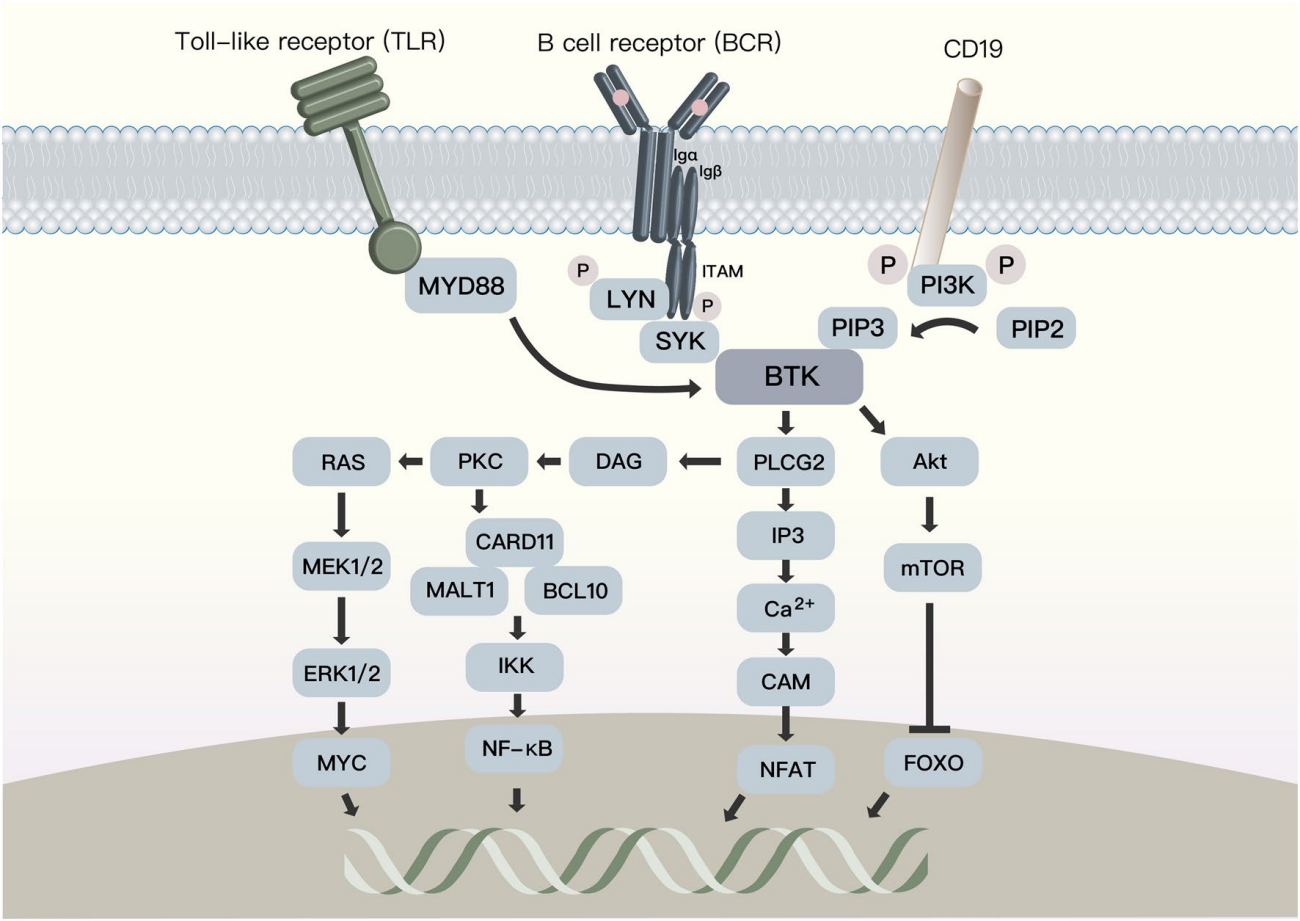
Upon antigen binding to the BCR, Src-family kinases such as LYN tyrosine kinase (LYN) and spleen tyrosine kinase (SYK) phosphorylate immunoreceptor tyrosine-based activation motif (ITAM) of Igα and Igβ, thereby recruiting spleen tyrosine kinase (SYK). SYK then phosphorylates and activates BTK. Subsequently, BTK phosphorylates phospholipase-Cg2 (PLCG2), and further initiates a series of downstream signaling pathways including nuclear factor kappa B (NF-kB), mitogen-activated protein kinase (MAPK), CaM and other pathways that promote cell proliferation and survival. In addition, BTK can also transmit various surface molecular signals such as Toll-like receptors (TLRs) that B cells communicate with the microenvironment; Tonic BCR: LYN also phosphorylates tyrosine residues in the cytoplasmic tail of the BCR co-receptor CD19, which countributes to the activation of phosphoinositol-3 kinase (PI3K) /AKT/mTOR signaling in antigen-independent manner

BCR is a transmembrane protein complex that controls B-cell fate from the beginning of its expression in the form of pro-BCR and pre-BCR and thus guides cell maturation, survival, apoptosis and the production of antibodies in plasma cells [8, 9]. BCR signaling is connected by a network of kinases and phosphatases that tune and amplify its activation. In general, BCR signaling pathways can be classified into two types: chronically activated BCR and tonic BCR [10]. Chronically activated BCR is an antigen-dependent process mainly utilizing the canonical nuclear factor-kB (NF-kB) pathway, MAPK/ERK pathways and ect. Conversely, tonic BCR maintains B cell survival through PI3K/AKT pathway by antigen-independent process [9, 11] (Fig. 1).

**Fig. 2:**
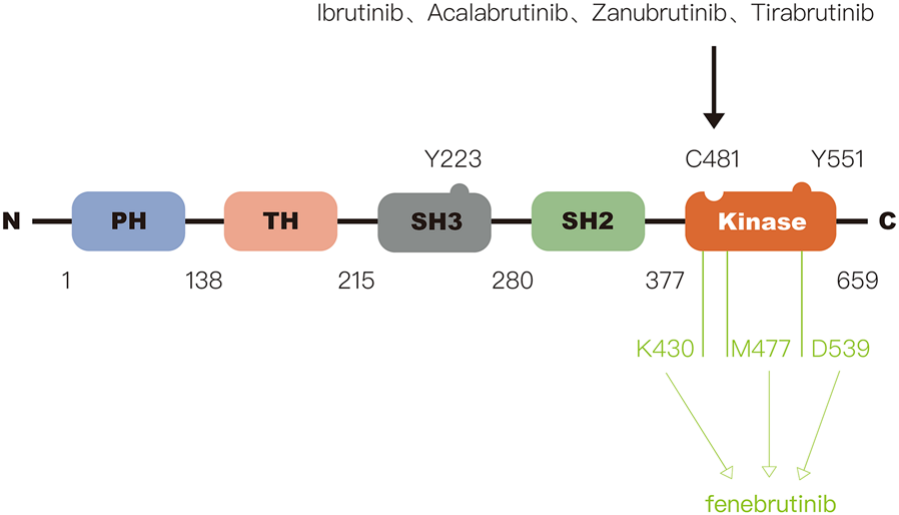
The structure of BTK. BTK protein includes 659 amino acids and 5 domains (PH, TH, SH3, SH2, Kinase domain). Among them, Y223 in the SH3 domain and Y551 in the kinase domain are two critical tyrosine phosphorylation sites. The covalent BTK inhibitors, including ibrutinib, acalabrutinib, zanubrutinib, and tirabrutinib, selectively bind to C481 residue in kinase domain. The non-covalent BTK inhibitors do not bind to C481. For example, Fenebrutinib forms hydrogen bonds with K430, M477, and D539 residues

BTK is a non-receptor intracellular kinase that belongs to the TEC family of tyrosine kinases, together with bone marrow-expressed kinase (BMX), redundant-resting lymphocyte kinase, and ITK. BTK has: (i) a kinase domain with enzymatic activity, (ii) SRC homology (SH) domains (including SH2 and SH3), (iii) a TEC homology (TH) domain, and (iv) an N-terminal pleckstrin homology (PH) domain [12, 13] (Fig. 2). BTK acts as a crucial component to couple BCR to more distal signaling, whose inactivation results in defects in B-cell development and function [14]. Upon BCR activation, BTK is recruited to the plasma membrane from cytoplasm by its PH domain binding phosphatidylinositol (3,4,5)-trisphosphate. At the plasma membrane, BTK is phosphorylated by SYK and SRC kinases at Y551 in the kinase domain and then autophosphorylates Y223 in its SH3 domain [14]. Phosphorylated BTK activates PLCG2 to further trigger a series of downstream signaling cascades.

Constitutive BCR or aberrant BTK activation usually lead to B-cell malignant transformation, which has been implicated in the pathogenesis of various BCLs. Moreover, malignant B cells often hijack normal BCR/BTK pathways to maintain their growth and survival [15]. BTKi was thus designed and developed successfully to target BCR/BTK signaling for the treatment of BCLs. However, since other kinases such as ITK, TEC, and BMX also harbour a corresponding residue in the ATP-binding site, a series of off-target effects, including bleeding, atrial fibrillation, and infection can, occur.

## First-generation and next-generation BTKis

Ibrutinib is a first-generation covalent irreversible BTKi that binds to C481 within the BTK active site and acts as a potent ATP competitive inhibitor, with a half-maximal inhibitory concentration of 0.5 nM. It can covalently inhibit other kinases, including ITK, TEC, EGFR, ErbB2, ErbB4, BMX, JAK3, and HER2 [16]. The off-target inhibitions of these kinases lead to a variety of adverse events. The most common adverse event in patients with CLL is infection (83%) that may be related to the inhibition of ITK in T cells and that of BTK in neutrophils and macrophages [17]. Bleeding and atrial fibrillation are among the frequent side effects of ibrutinib, the latter inhibiting BTK and TEC kinases, resulting in impaired platelet activation and cardiac PI3K-Akt pathway downregulation [18, 19]. Diarrhoea was reported in 52% of patients with CLL, who were treated with ibrutinib, due to the inhibition of EGFR by the latter [17, 20]. To reduce these off-target effects and improve tolerability, next-generation BTKis, such as acalabrutinib, zanubrutinib, and orelabrutinib, have been designed and developed to covalently attach to BTK via C481, exhibiting greater selectivity for BTK and having fewer off-target effects than ibrutinib. Non-covalent BTK inhibitors, such as pirtobrutinib, vecabrutinib, and fenebrutinib, which do not bind C481, are reported to have fewer off-target toxicity, thus providing a promising effective option for patients with B-cell lymphoma, especially those with BTK C481 mutations. Fenebrutinib does not inhibit EGFR or ITK; thus, it may greatly alleviate diarrhoea and rash due to EGFR inhibition and preserve NK cell-mediated ADCC [21]. Adverse effects of the various BTKis with relative frequencies are shown in Table 1. Recently, they have been well studied and have shown manageable safety profiles and efficacy [22]. Furthermore, the HSP90 inhibitor SNX-5422, which is also a BTK protein degrader, has been explored for BTK inhibitor-resistant CLL [23].

**Table 1:** The adverse effects and relative frequencies of different BTKi in B-cell malignancies

BTK inhibitors	Disease state	Adverse Effects						
		Infection (grade ≥ 3)/pneumonia	Neutropenia	Diarrhea	Hypertension	Hemorrhagea/Major bleeding	Atrial fibrillation and flutter	References
Ibrutinib	R/R CLL	Grade ≥ 3 11%/19%	16%	55%	22%	44%/1%	3%	[114–116]
	TN CLL	Grade ≥ 3 48%/4%	4%	68%	22%	major bleeding 4%	6%	[115–117]
	R/R MCL	Pneumonia 13%	17%	< 5%	5%	Grade ≥ 3 6%	Grade ≥ 3 7%	[118]
	WM	Pneumonia 12%	13%	32%	16%	hematuria 10%	15%	[119]
Zanubrutinib	B-cell malignancies (33%WM, 29%CLL/SLL, 19%MCL)	Grade ≥ 3 27%/21%	36%	23%	12%	55% / 4%	3%	[120]
Acalabrutinib	B-cell malignancies	Grade ≥ 3 (18%)/9%	12%	37%	8%	4%	4%	[121]
orelabrutinib	B-cell malignancies	Grade ≥ 3 (15%)/2%	29%	7%	–	< 1%	< 1%	[122]
Pirtobrutinib	R/R B-cell malignancies	7%	13%	17%	–	< 5%	< 1% (unrelate to pirtobrutinib)	[123]
Fenebrutinib	R/R B-cell malignancies	Grade ≥ 3 (17%)/4%	4%	29%	–	8%	–	[124]
Vecabrutinib	R/R B-cell malignancies	–	25%	–	–	–	–	[125]

## Effects of BTK and BTKi in innate immunity

BTK is widely expressed in innate immune cells and plays a pivotal role in innate immunity [24]. It is indispensable for the development and maturation of neutrophils [25]. Neutrophil count is decreased in patients with X-linked agammaglobulinaemia due to growth arrest [26, 27]. Additionally, with exposure to ibrutinib, multiple functions of neutrophils, such as the production of reactive oxygen and engulfment of *Aspergillus*, are significantly impaired, which severely affects the innate immune response [28].

BTK not only induces TLR and Fc receptor signalling pathways but also regulates NLRP3-inflammasome activity in macrophages, monocytes, and dendritic cells (DCs) [29–31]. Macrophages can phagocytise and kill pathogens, acting as the first line of defence against fungal infections. Several studies have shown that exposure to ibrutinib and acalabrutinib can inhibit phagocytosis and secretion of inflammatory factors in macrophages and monocytes, thereby increasing susceptibility to infection [32–34]. In addition, ibrutinib suppresses the secretion of CXCL13, which can attract and protect CLL cells from tumour-associated macrophages or nurse-like cells in the bone marrow of patients with CLL [35]. Importantly, myeloid-derived suppressor cells (MDSCs) with BTK expression can be inhibited by ibrutinib, thereby potentially enhancing the efficacy of cancer vaccines [36]. Zou et al. [37] found that zanubrutinib could downregulate the expression of PD-L1 in MDSCs and restore the immune response. DCs are potent antigen-presenting cells that play an important role in initiating, regulating, and maintaining the immune response. By triggering the secretion of inhibitory factors in DCs, hepatocyte growth factor- and T-cell immunoglobulin and mucin protein-3-mediated BTK activity inhibits the NF-κB pathway and consequently blocks the activation and maturation of DCs [38, 39]. Natarajan et al. [40] had reported that ibrutinib-treated DCs can prompt T-cell proliferation and Th17 response.

BTK is a crucial regulator of the functions of NK cells, since BTK-deficient NK cells have impaired cytotoxic activity [41]. Ibrutinib significantly suppresses NK cell-mediated cytotoxicity and ADCC that cannot be reversed by lenalidomide, a potential sensitiser to anti-CD20 therapy in MCL cell lines [42, 43]; however, acalabrutinib, orelabrutinib, zanubrutinib, and fenebrutinib do not influence the NK cell effector function [44, 45], probably due to their weaker or even no off-target inhibition (e.g., fenebrutinib for ITK) [21, 44]. Rituximab exerts an antitumour effect mainly through NK cell-mediated ADCC. Over the past few decades, CD20 monoclonal antibodies have been empirically added to other therapies by clinicians to improve the therapeutic efficacy in BCL. In ECOG E1912 trial, the combination of ibrutinib and rituximab versus fludarabine, cyclophosphamide, and rituximab significantly prolonged progression-free survival and overall survival and alleviated therapy toxicity in untreated patients with CLL/SLL [46]. However, a randomised single-centre study comparing ibrutinib plus rituximab with single-agent ibrutinib in patients with CLL showed that the combination therapy neither improved progression-free survival nor overall response rate (ORR) [47]. Preclinical data showed the combination of orelabrutinib and rituximab to enhance NK cell-induced ADCC and exert a synergistic antitumour effect in BCL [48]. Based on these findings, acalabrutinib plus obinutuzumab was considered to show high and durable responses in treatment-naïve patients and in those with R/R CLL [49, 50]. A phase-3 study reported by Sharman et al. [51, 52], combining ublituximab with ibrutinib, showed a higher ORR (83% vs 65%) than that obtained in the group of patients with R/R high-risk CLL receiving ibrutinib alone; similar results were found in patients with R/R MCL. Combination of ublituximab and ibrutinib reduced the lymphocytosis induced by single-agent ibrutinib treatment, thereby partially explaining why the stronger ADCC effect of ublituximab was not affected by ibrutinib. Natural killer T (NKT) and γδ T cells bridge innate and adaptive immunity. In CLL, NKT cells indirectly hinder tumour cell survival and are important mediators of tumour surveillance and prognosis [53]. However, several studies have demonstrated that NKT- and γδ T-cell counts decrease in patients with CLL undergoing ibrutinib treatment [54, 55].

BTK and ITK are key regulators of Fc receptor signalling in mast cells; they have been implicated in the regulation of FcγRI-mediated responses, such as degranulation and cytokine production in mice [56]. In human cells, ibrutinib inhibits these functions [57].

Recent studies have shown that ibrutinib and acalabrutinib can prevent sialic acid-binding immunoglobulin-like lectin (Siglec)-8-induced eosinophil and basophil death [58]. Eosinophils and basophils are effector cells that play a crucial role in the defence against microbial infections. Recently, they have also been recognised as being related to COVID-19 [59]. Therefore, the relationship between BTKis and cell types would require further investigation.

## Effects of BTK and BTKi in adaptive immunity

In adaptive immunity, both T and B lymphocytes play key roles in the antitumour immune response via specifically recognising tumour antigens. Moreover, they can induce an immune response to infection [60]. An in-depth understanding of the biological effects of BTKis on these cells will help discover better targeted drugs and combination strategies.

### Effects on T cells

In CLL, immune functions are dysregulated due to profound defects in T-cell functions; an abnormal T-cell compartment may be related to disease activity or previous antitumour treatment [61]. Previous studies have shown that ibrutinib can increase the T-cell repertoire diversity in CLL, indicating that ibrutinib therapy prompts cellular immune reconstitution [62, 63]. A comprehensive summary of the effects of ibrutinib on T cells in CLL was presented by Mhibik et al. [64]. With respect to the changes in T-cell numbers, including major subsets, Long et al. [65, 66] established that CD4+ and CD8+ T-cell numbers are remarkably increased after 8 weeks of ibrutinib treatment, whereas the expression of programmed death-1 (PD-1) and cytotoxic T-lymphocyte-associated antigen-4 (CTLA-4) in T cells was reduced by ibrutinib and acalabrutinib. However, the cells significantly decreased in number over time during ibrutinib and zanubrutinib treatment [35, 37, 67, 68]. Tirabrutinib possibly has no effect on T-cell function [69]. Indeed, accumulating evidence suggests that the decline in the number of T cells may stay in step with the receding tumour burden [61, 70]. Unsurprisingly, patients with R/R CLL have higher T-cell numbers than untreated patients and non-progressive patients [61]. Therefore, the addition of ibrutinib to PD-L1 inhibitors has a synergistic effect compared to PD-L1 inhibition alone in animal models of lymphoma [71]. Previous trials have elucidated that activity of the combination of pembrolizumab or nivolumab with ibrutinib is limited in patients with CLL, although it is promising in patients with Richter transformation [72, 73]. Furthermore, BTK seems to be expressed in T cells, according to one report, especially in effector/memory T cells, and plays an important role in T-cell activation, indicating an on-target effect of BTKi on T cells [74]. In addition, ibrutinib has been shown to induce skewing towards Th1 cells and decrease in Th2 and Th17 cell numbers by inhibiting ITK [75]. However, acalabrutinib and zanubrutinib do not change Th1/Th2 cell numbers [37, 65], possibly because of their weak off-target effects on ITK.

Anti-CD19 CAR-T therapy has produced durable and complete responses in R/R BCL. However, the complete response rates are still below 50% in patients with CLL, which may be related to T-cell dysfunction [76]. Given the effects of BTKi on T cells, as mentioned above, emerging evidence indicates that pre-treatment with ibrutinib before apheresis can reverse T-cell dysfunction and benefit CAR-T-cell production, which may be used as a bridging therapy before CAR-T therapy [77]. Moreover, ibrutinib or acalabrutinib, in combination with CAR-T, can increase the number and function of T cells, promote the engraftment and expansion of CAR-T cells, improve the antitumour efficacy of CAR-T cells, and reduce cytokine release syndrome (CRS) in patients with CLL and MCL [3, 78–80]. Compared to CAR-T alone, its combination with ibrutinib improved the ORR from 56 to 83% in patients with R/R CLL, thus demonstrating promising therapeutic potential in BCL [3, 78–80]. Acalabrutinib does not inhibit ITK activity, although it potentiates CAR-T therapy, hence suggesting that other mechanisms may be involved in the acalabrutinib-mediated regulation of microenvironment. However, ibrutinib and acalabrutinib are irreversible receptor tyrosine kinase inhibitors that may inhibit CAR-T-cell proliferation and expansion [81]. In contrast to irreversible BTKi, the reversible, non-covalent BTKi vecabrutinib combined with CAR-T-cell treatment resulted in sustained antitumour activity and potentiated CAR-T-cell proliferation in a mouse JeKo-1 lymphoma xenograft model [81]. RNA sequencing of activated CD19 CAR-T-cells revealed significant upregulated expression of multiple genes related to PI3K/AKT and Th1 pathways [81]. Therefore, the synergistic effects of different BTKis on CAR-T cells should be explored further. A recent study reported that T cells from patients with CLL who were treated with ibrutinib when combined with BiAbs had more significant antitumour effects than T cells from those not treated with ibrutinib [82]. The same conclusion was further confirmed by the enhanced cytotoxic activity of T cells in patients treated with ibrutinib [83]; however, the underlying mechanisms remain to be fully elucidated. Another study found that A-319, a CD19/CD3 BiAb, in combination with ibrutinib, enhanced the antitumour efficacy and inhibited CRS, possibly by regulating macrophage-induced angiogenesis [84].

### Effects on B cells

Hypogammaglobulinaemia occurs in most patients with CLL, especially those with progressive disease, with a frequency varying from 20 to 70% [85–88]. Several studies have shown that hypogammaglobulinaemia correlates with the risk of infection in CLL [88, 89]. Normal B-cell count appears to be increased during ibrutinib treatment while still being aberrantly lower than that in healthy individuals, which may be correlated with serum BAFF levels [90]. BTK plays a crucial role in B-cell development, maturation, and immunoglobulin (Ig) production. Although B cells and Igs are usually absent in patients with BTK-deficient XLA, a decrease in IgG levels is not observed over a short-term ibrutinib treatment [90]. IgA level continues to increase with the long-term administration of ibrutinib and acalabrutinib in patients with CLL [90, 91]. The results suggest that BTKis can cause partial humoral and cellular immune reconstitution in CLL. The immunomodulatory effects of BTKi are summarised in Table 2.

**Table 2:** Immunomodulatory effects of BTKi

	Cell type	Impact of BTKi on cell number				
		Ibrutinib	Acalabrutinib	Zanubrutinib		
Innate immune system	Neutrophils	Decrease	–	–	Inhibit BTK	[25–28]
	Macrophages	Decrease	Decrease	–	Inhibit BTK by TLR, FcR and NLRP3-inflammasome	[29–31]
	Monocytes/DC	Decrease	Decrease	–	Inhibit BTK by TLR, FcR and NLRP3-inflammasome	[32–34, 37–40]
	TAM (nurse-like cells)	Decrease	–	–	Unknown	[35]
	MDSC	Decrease	–	Decrease	Inhibit BTK	[36–39]
	NK	Decrease	–	No change	Inhibit ITK	[41–43]
	NKT	Decrease	–	–	Unknown	[53–55]
	γδ T cells	Decrease	–	–	Unknown	[54, 55]
	Mast cells	Decrease	–	–	Inhibit BTK, ITK and TEC	[56, 57]
	Basophils/eosinophils	–	–	–	Prevent (Siglec)-8–induced cells death by inhibting BTK	[58]
Adaptive immune system	Tcells					
	CD4^+^	Increase first then decrease	No change	Increase first then decrease	The receding tumor burden and ITK	[35, 37, 62, 63, 65–68]
	CD8^+^	Increase first then decrease	No change	Increase first then decrease	The receding tumor burden and ITK	[35, 37, 62, 63, 65–68]
	Th1	Increase	No change	No change	Mediated by RLK	[37, 65, 74, 75]
	Th2	Decrease	No change	No change	Inhibit ITK	[37, 65, 74, 75]
	Th17	Decrease	No change	No change	Inhibit ITK	[37, 65, 74, 75]
	Tregs	Decrease	–	Decrease	Inhibit ITK	[37, 65, 74, 75]
	B cells					
	IgA production	Increase	Increase	–	Unknown	[90, 91]
	IgG production	Decrease	–	–	Unknown	[90]

## Effects of BTKis on pathogenic infections

Infections increase the morbidity and mortality of patients with CLL and constitute a major reason for treatment discontinuation [92]. The increased risk of infections seems to correlate with inherent immune defects in CLL, such as the above-mentioned abnormal T-cell subsets, hypogammaglobulinaemia, and previous anticancer treatment [92].

**Fig. 3:**
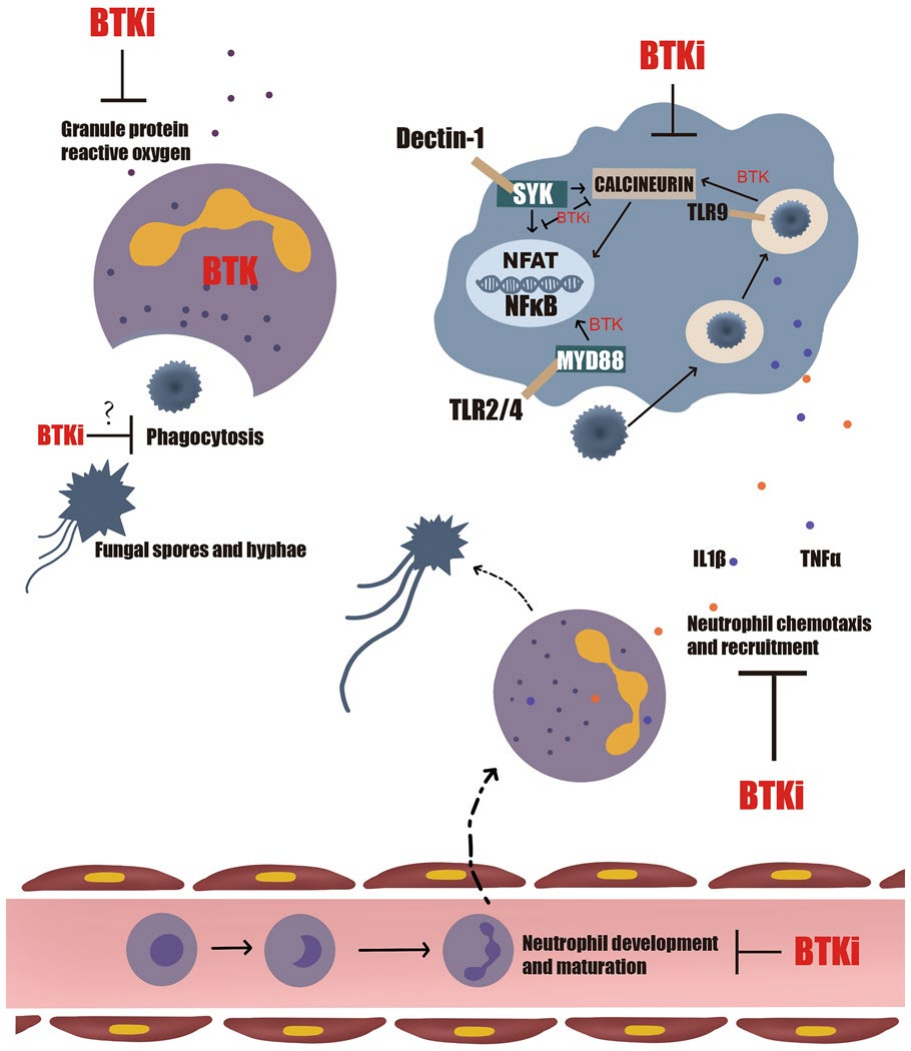
When fungal infection happens, neutrophils was recruited to the sites of inflammation by cytokines and then release granule proteins and reactive oxygen. BTKis seem to block their release. Additionally, BTK plays a crucial role in neutrophils development and maturation, which can also be inhibited by BTKis. Macrophages also mediate the recruitment of neutrophils by releasing IL1β and TNFα. In addition, macrophages can eliminate fungus by phagocytosis. TLR2/4 and Dectin-1 can recognize β-glucans, chitins and mannans (PAMPs) of fungus and then induce downstream signaling cascades via BTK including NFκB and nuclear factor of activated T-cells (NFAT). However, BTKi not only impair the phagocytosis of macrophages, but also inhibit cytokine release and disrupt the signal transduction

Although ibrutinib has exhibited excellent efficacy against CLL, several studies have reported increased number of infectious events during ibrutinib treatment, especially in the first 6 months [33]. Consistent with these findings, Sun et al. [90] found that although IgG levels declined, IgA levels increased after 6 months with ibrutinib treatment, and this result was related to low infection rates. Approximately 56% of patients with haematologic malignancies have been reported to experience at least one infectious event of any grade during single-agent ibrutinib treatment. Grade 3–4 infectious events accounted for 26% of all events, and half of them were pneumonia [93]. In another study, 5% of patients had opportunistic infections (grade ≥ 3), including *Aspergillus fumigatus*, *Pneumocystis jirovecii,* and *Mycobacterium tuberculosis* infections, *Aspergillus* causing the most common fungal infection in CLL [94]. However, the incidence of these fungal infections was extremely low in WM with ibrutinib treatment, suggesting that features of the disease itself play a role, beyond the effects of ibrutinib, such as in immunodeficiency [95]. In a recent study, ibrutinib and acalabrutinib were found to potentially inhibit the antifungal effect of platelets, such as the ability to adhere to conidia, and thus increase susceptibility to fungal infection [96]. Similarly, another study demonstrated that ibrutinib inhibited bacteria (*Staphylococcus aureus* and *Escherichia coli*)-induced platelet activation via an FcgRIIA/aIIbb3-dependent pathway, resulting in impaired platelet-mediated clearance of bacteria [97]. Other infections, such as gastrointestinal, genitourinary, and skin infections, have also been observed in patients with ibrutinib treatment. The rate of infection of grade ≥ 3 was reported in over 40% of patients with R/R CLL/small lymphocytic lymphoma [98], but the rate declined to only 10–21% in a previously untreated group [99–101]. The increased infection rate can be attributed to the impairment of innate and adaptive immunity by the BTKi via inhibition of BTK (on-target effect) and ITK (off-target effect) signalling pathways. The role of a BTKi in the immune cell response (mainly neutrophils and macrophages) to fungal infection is shown in Fig. 3. However, the next-generation BTKis acalabrutinib and spebrutinib, despite being endowed with greater selectivity and no off-target effect on ITK, can also inhibit macrophages and the neutrophil-induced antifungal response to predispose the patient to infection [32]. Parallel to these results, 23% and 12% of infection complications (grade ≥ 3) were observed in two phase II studies in patients with R/R CLL treated with acalabrutinib and zanubrutinib, respectively [102, 103]. Therefore, more studies should be conducted to elucidate the differences between different BTKis and infection susceptibility and to understand the related mechanisms.

## BTKi and COVID-19

The COVID-19 pandemic, caused by a beta-coronavirus named SARS-CoV-2, has spread worldwide since its emergence in 2019 and is associated with high morbidity and mortality. More than one-third of the patients with COVID-19 develop acute respiratory distress syndrome and require intensive care unit (ICU) admission [104]. Severe T-cell dysfunction, macrophage activation, and high levels of cytokines and chemokines have been observed in the bronchoalveolar lavage fluid of these patients with severe/critical disease [105], suggesting that targeting immune responses, including excessive host inflammation, may contribute to COVID-19 treatment.

**Fig. 4:**
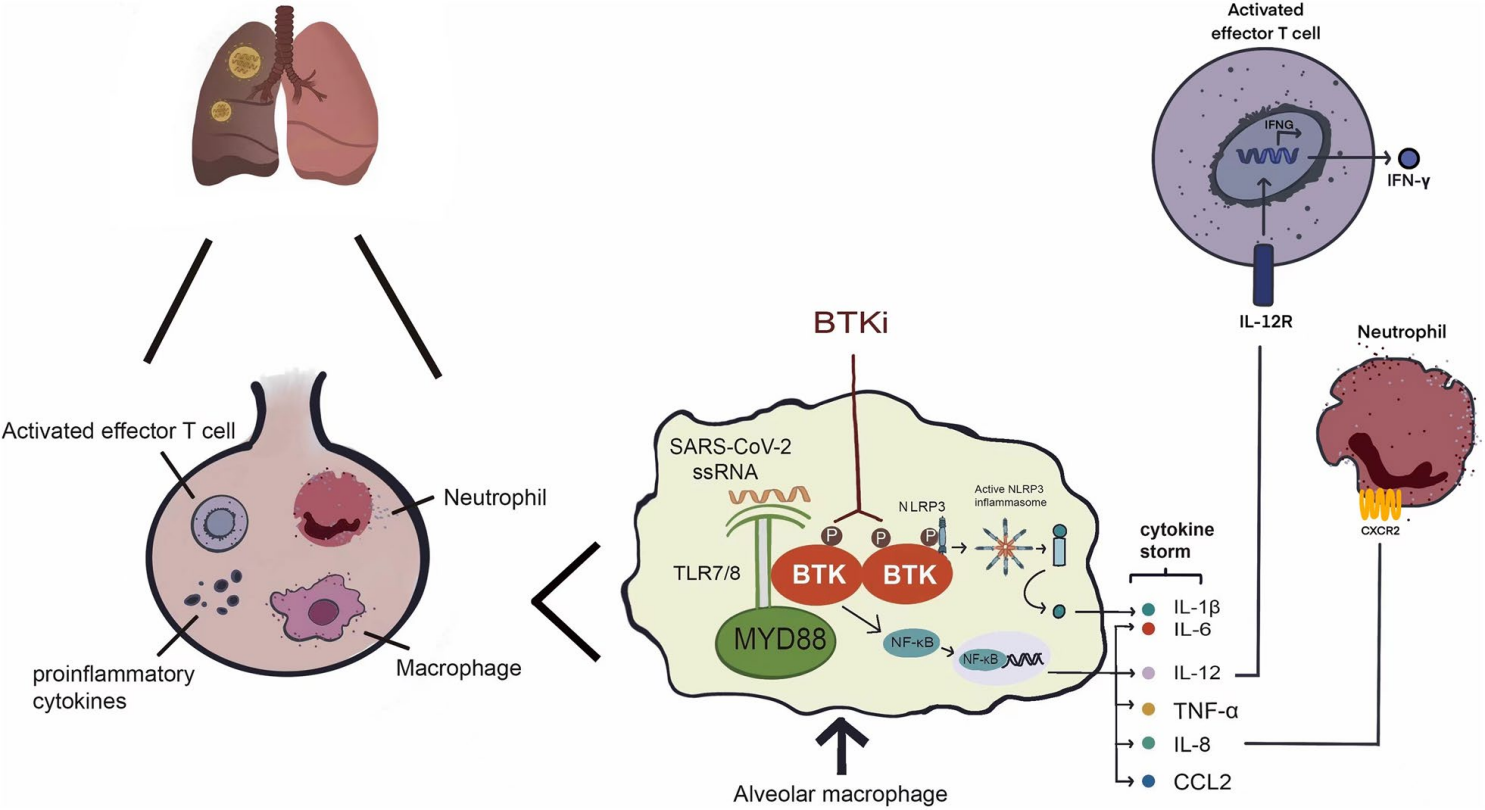
When SARS-CoV2 enters the respiratory tract and causes an infection, alveolar macrophages may engulf viral particles or cellular debris. Viral single-stranded RNA (ssRNA) binds to Toll-like receptor7/8 (TLR7/8) and then activates Bruton tyrosine kinase (BTK) and myeloid differentiation primary response 88 (MYD88). For one thing, activation of the BTK-dependent nuclear factor kappa B (NF-κB) pathway leads to the production of a series of pro-inflammatory factors and chemokines, which are called cytokine storms. Among these, IL-8 can recruit more neutrophils in the late phase of severe coronavirus disease 2019(COVID-19 infection. BTKi can inhibit TLR-dependent NF-κB signaling pathway, thereby preventing cytokine production. For another, during severe COVID-19, the accumulated NLR family pyrin domain containing 3(NLRP3)inflammasome is phosphorylated by BTK, thus promoting its oligomerization and assembly into an inflammasome (pro-IL-1β to mature IL-1β). BTKis inhibit inflammasome-mediated process

Given the immunomodulatory effect of BTKis, clinical studies on their effects in patients with BCL and COVID-19 are still ongoing. One study reported that an 81-year-old patient with WM and COVID-19 required non-invasive ventilation in the ICU once ibrutinib was discontinued, and respiratory symptoms improved significantly after resuming ibrutinib treatment [106]. Moreover, acalabrutinib, zanubrutinib, and spebrutinib were demonstrated to mitigate the lung injury mediated by a cytokine storm by inhibiting the BTK-dependent NF-κB pathway, normalising T lymphocytes, and even exerting antiviral effects as ligands in COVID-19 [107, 108]; the specific mechanism is illustrated in Fig. 4. Abivertinib, a third-generation BTKi, has shown excellent efficacy in hospital in-patient admission for symptomatic COVID-19, and related clinical research is currently ongoing. To the best of our knowledge, CLL is characterised by advanced age and immune dysfunction, making the patients more susceptible to COVID-19 and its complications. Therefore, we aimed to determine whether patients with CLL who were treated with BTKi and diagnosed with symptomatic COVID-19 had a better prognosis. According to a report by Mato et al. [109], CLL-directed therapy (mainly ibrutinib) did not affect the survival of patients with COVID-19, even if ibrutinib was often interrupted once COVID-19 was diagnosed. Conversely, ibrutinib exhibited a protective effect in a study by Scarfò et al. [110]. Further investigations are, therefore, warranted to ascertain the role of BTKis and COVID-19 in CLL and other BCLs.

As the most powerful weapon against infection, vaccines have been developed against SARS-CoV-2. However, according to recent reports, the vaccine response seemed to be suboptimal in patients with CLL, especially those under BTKi treatment and/or with R/R disease [7, 111]. This result may be attributed to the impaired humoral immune response to COVID-19 vaccination due to BTKis and R/R patients being more immunocompromised than treatment-naive patients [111, 112]. In contrast, 75% of ibrutinib-treated patients with CLL produced a cellular response to vaccination, although they had a severely compromised humoral immune response [113]. This result was not surprising, since ibrutinib is capable of restoring the T-cell number and function. However, the severe disease burden impairs the ability to generate cellular immune responses in patients with CLL [113]. In addition, the humoral response to adjuvant recombinant hepatitis B (HepB-CpG) vaccine was lower in patients on a BTKi than in treatment-naïve patients (3.8% vs. 28.1%); however, intriguingly, the recall response to recombinant zoster vaccine did not differ across the groups [112].

## Conclusions and perspectives

BTKi exerts excellent antitumour efficacy while reshaping the immune system, hence providing a rationale for well-designed combination therapies. More selective BTKis, such as acalabrutinib, orelabrutinib, and fenebrutinib, in combination with anti-CD20 antibodies, are well tolerated and yield durable responses by enhancing NK cell-induced ADCC. Ibrutinib can enhance the expansion and degranulation ability of T cells and reduce the expression of PD-1 and CTLA-4. Therefore, the combination of BTKi with CD19 CAR-T cells, BiAbs, or checkpoint blockade warrants further exploration. The effects of ibrutinib on the function and number of innate immune cells such as neutrophils, MDSCs, mast cells, monocytes and macrophages are mainly due to the inhibition of BTK and Tec kinases, which is also validated by studies on acalabrutinib and zabrutinib. In addition, ibrutinib modulates NK cells by inhibiting ITK, whereas acalabrutinib and zanubrutinib have no such effect due to a weaker inhibition of ITK. To the best of our knowledge, there is no relevant reports revealing the functional and mechanistic roles of these three BTKis in regulation of NK-T cells, γδ T cells, eosinophils and basophils. With regard to adaptive immune cells, ibrutinib is shown to alter T-cell (including CD4+, CD8+ T cells, Th2, Th17, Tregs) number and function primarily through ITK inhibition, zabrutinib and acalabrutinib yet acalabrutinib have no such effect due to a weaker inhibition of ITK. In addition, the mechanism by which ibrutinib and acalabrutinib increase IgA levels and ibrutinib decreases IgG are unclear (refer to Table 2).

The impairment of the immune system caused by BTKis aggravates CLL defects. An increasing number of infections (particularly fungal infections) and pneumonia have been reported in patients treated with ibrutinib, especially in R/R patients. The infection rate is the highest in the initial months of ibrutinib therapy and declines with decreasing tumour burden [90]. With respect to COVID-19, BTKis appeared to dampen the cytokine storm by inhibiting the monocyte/macrophage activation induced by COVID-19 and improving the survival of patients with CLL [108, 110], which raises the prospect of BTKis being useful for other diseases related to macrophage activation; long-term research in this field would be worth exploring. BTKis have been confirmed to abrogate the immune response to novel antigens, suggesting that a patient-tailored vaccination approach should be adopted in patients with CLL, according to disease status and previous treatment, such as in the early stages of the disease or before BTKi administration. Further investigations on the effects of BTKis on the immune system and potential combination therapy should be explored to provide the best clinical practice guidance to clinicians dealing with adverse events such as infections.

## Data Availability

Data sharing is not applicable to this article as no datasets were generated or analysed during the current study.

## Abbreviations

ADCC: Antibody-dependent cellular cytotoxicity
BCR: B-cell receptor
BiAb: Bispecific antibody
BMX: Bone marrow-expressed kinase
BTKi: Bruton tyrosinase kinase inhibitor
CAR-T: Chimeric antigen receptor-T cell
CLL: Chronic lymphocytic leukaemia
CTLA-4: Cytotoxic T-lymphocyte-associated antigen-4
CRS: Cytokine release syndrome
EGFR: Epidermal growth factor receptor
ICU: Intensive care unit
Ig: Immunoglobulin
ITK: IL-2-inducible T-cell kinase
MDSC: Myeloid-derived suppressor cell
NK: Natural killer
NKT: Natural killer T cells
NLRP3: NACHT, LRR, and PYD domain-containing protein 3
ORR: Overall response rate
PD-1: Programmed death 1
PD-L1: Programmed death-ligand 1
PI3K: Phosphoinositide 3-kinase
R/R: Relapsed/refractory
SH: SRC homology
TCR: T-cell receptor
TH: TEC homology
TLR: Toll-like receptor
TME: Tumour microenvironment
WM: Waldenstrom macroglobulinaemia
